# Wrist Immobilization after Surgical Decompression of the Median Nerve in Carpal Tunnel Syndrome: A Systematic Review

**DOI:** 10.1055/s-0044-1785665

**Published:** 2024-05-19

**Authors:** Thiago Moreth da Silva Barbosa, Ítalo Carvalho Ferraz

**Affiliations:** 1 Departamento de Ortopedia e Traumatologia, Hospital Getúlio Vargas, Recife, PE, Brasil

**Keywords:** carpal tunnel syndrome, median nerve, peripheral nerve injuries, wrist injuries

## Abstract

**Objective** The most common compressive neuropathy of the upper limbs is carpal tunnel syndrome (CTS). Historically, there has been a tendency to apply immobilization in the postoperative period, a practice that has decreased in recent years. This review aims to assess whether there is scientific evidence to justify the use of immobilization in the postoperative care of CTS decompression.

**Methods** The following databases were used: Biblioteca Virtual em Saúde (BVS), PubMed National Library of Medicine – (NLM), Cochrane Library, Scientific Electronic Library Online (SciELO), and EMBASE. The following inclusion criteria were used: 1) discussion of the postoperative period of median nerve decompression surgery in CTS; 2) comparison of results after surgical decompression in CTS between wrist immobilization or local dressing; 3) all languages, regardless of the year of publication; and 4) all types of publications. The following exclusion criteria were used: 1) studies that did not evaluate the postoperative period of CTS decompression; 2) lack of evaluation of the outcome related to the application of local dressing or some form of wrist immobilization after the surgical decompression procedure; and 3) repeated publications.

**Results** The literature search resulted in 336 relevant publications. In the end, 18 publications were chosen. Systematic reviews, randomized clinical trials, and cross-sectional studies were found.

**Conclusions** Due to the scarcity of evidence supporting the use of immobilization coupled with the higher costs associated with the practice, it has become less and less frequent in recent decades.

**Clinical relevance** In the literature, two approaches to postoperative care for CTS decompression are described: immobilization or just local dressing. According to the available scientific evidence, it is worth evaluating which one is better.

## Introduction

The most common compressive neuropathy of the upper limbs is carpal tunnel syndrome (CTS). The most frequent symptom is paresthesia, especially at night, in the region innervated by the median nerve. Surgical treatment is preferable in cases in which conservative therapy fails. Surgical decompression can be open or endoscopic. 1 Historically, there was a tendency to apply immobilization in the postoperative period, a practice that has decreased in recent years. 2 3 4 Immobilization would theoretically have the advantage of avoiding the bowstring effect of the flexor tendons and promoting analgesia. 5 However, more recent studies do not support this idea.

The literature describes two courses of action in the postoperative period of CTS decompression: immobilization or local dressing only. According to the available scientific evidence, it is worth evaluating which one is better.

The aim of the present study is to evaluate the outcomes after CTS decompression surgery when the wrist is immobilized and when only a bandage is applied and to compare them.

## Material and Methods

### Research Strategy

An active search for articles in the literature was carried out on May 6, 2023. The search used the following databases: Virtual Health Library (VHL), PubMed National Library of Medicine (NLM), Cochrane Library, Scientific Electronic Library Online (SciELO), and EMBASE. The descriptors used, together with the Boolean operators, were: “ *Carpal Tunnel Syndrome* [Mesh] OR *carpal tunnel syndrome* [tw] OR *carpal tunnel release* [tw] AND *Postoperative Care* [Mesh] OR *postoperative* [tw] OR *postoperative care* [tw] AND *Restraint, Physical* [Mesh] OR *immobilization* [tw] OR *splint* * [tw] AND *Bandages* [Mesh] OR *dress* *[tw] OR *bandage* *[tw]. A cross-reference search was also done in the databases to find articles not initially identified.

The search strategy for each database is detailed in the appendices at the end of the paper.

### Selection Criteria

The following criteria were used to include the studies: 1) discussion of the postoperative period of median nerve decompression surgeries in CTS; 2) comparison of outcomes after surgical decompression in CTS between wrist immobilization or local dressing alone; 3) all languages, regardless of the year of publication; and 4) all types of publications.

The following exclusion criteria were listed: 1) studies that did not evaluate the postoperative period of CTS decompression; 2) absence of an evaluation of the outcome related to the conduct of local dressing or some form of wrist immobilization after the surgical procedure; and 3) repeated publications.

### Data collection

Two researchers carried out the data collection process. Initially, the titles and abstracts of each publication were analyzed independently, after which any inconsistencies were discussed, and a consensus was reached. Subsequently, each publication was read in full independently, and meetings were held to decide which studies should be included and excluded, reaching a mutual consensus.

### Level of Evidence

The studies were classified from I to V according to the hierarchical level of evidence. 6 The Preferred Reporting Items for Systematic Reviews and Meta-Analyses (PRISMA) guidelines were used in this review for the search, data extraction, and analysis of results. 7

## Results

**Fig. 1:**
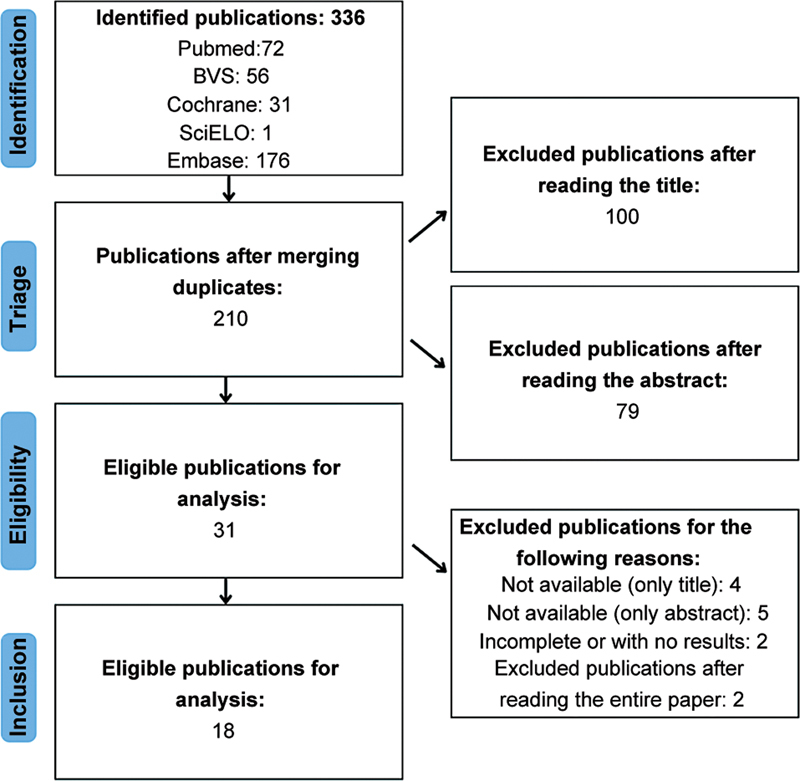
Preferred Reporting Items for Systematic Reviews and Meta-Analyses (PRISMA) diagram to illustrate the search process according to this study's inclusion and exclusion criteria.

The literature search resulted in 336 relevant publications. After removing duplicates, 210 articles were obtained. Subsequently, after analyzing the titles and abstracts, 179 studies were excluded, resulting in 31 eligible articles. After a complete reading of the text, four articles were eliminated due to the availability of only the title on digital platforms, and another five were excluded due to the availability of only the abstract. Two articles were eliminated because they did not present the results (only the body of the work was available, as it had not been finalized). Two other studies were excluded after a complete reading of the text because they did not fit the study's objective. In the end, 18 publications were listed. Fig. 1 illustrates the research process.

### Systematic Reviews - Level of Evidence I

A systematic review by Ashworth found randomized clinical trials (RCTs) comparing immobilization versus non-immobilization groups after CTS decompression. At 6 months, no differences were found in grip and pinch strength between patients in the 2 groups. Another RCT showed that return to work was slower in the immobilized group (27 days versus 17), and there were higher rates of pillar pain and scar tenderness after 1 month. 8

Similarly, Huisstede et al. 9 found five RCTs that showed no evidence of benefit from immobilization. Grip and pinch strength, analgesia, and functionality were comparable in both groups.

A systematic review by Peters et al., 10 which included 20 trials with 1,445 participants, found little scientific evidence in favor of using postoperative immobilization. Later, in 2015, Peters et al. 11 updated the previous review from 2013. They included 22 trials and 1,521 patients. The results remained the same.

Table 1 shows the systematic review articles found.

**Table 1:** Level I evidence studies

Authors	Title	Level of evidence
Ashworth 2010	Carpal tunnel syndrome	RS (I)
Huissttede et al. 2010	Carpal tunnel syndrome. Part II: effectiveness of surgical treatments-a systematic review	RS (I)
Peters et al. 2013	Rehabilitation following carpal tunnel release	RS (I)
Peters et al. 2016	Rehabilitation following carpal tunnel release	RS (I)

### RCT - Level of Evidence II

An RCT evaluated 40 patients and 43 open CTS decompressions, comparing the 2-week postoperative period between a group of 26 who received a splint and a group of 17 who received only a bulky dressing. No beneficial effect was observed in that group when evaluating the objective parameters of grip strength, pinch strength, complications, range of motion in the hand and fingers, and the subjective parameter of personal satisfaction. 5

Another RCT evaluated 50 patients, divided equally into 2 groups. One group received a splint after the decompression procedure for CTS, while the other group was allowed free range of motion in the immediate postoperative period. In the immobilized group, higher rates of pain, scar tenderness, stiffness, and longer recovery time were observed. 12

In one publication, Finsen et al. 13 analyzed 82 wrists that had undergone CTS decompression and were randomly divided into a group without immobilization and a group with postoperative immobilization for 4 weeks. No benefits or harm related to the practice were found. In another publication, 52 patients were divided equally into 2 groups: 26 who received immobilization for 2 weeks after CTS decompression and 26 patients without postoperative immobilization. Both groups had similar outcomes, including two-point discrimination. It was concluded that the immobilized group was not superior to the control group. 14

Bhatia et al. 15 showed, from an RCT with 102 patients, that using a volar splint in the postoperative period was not correlated with any analgesic effect compared to the control group. After 50 open CTS decompressions, half received only a dressing, and the other half received a volar splint for 2 days. These patients were followed up for 3 months after surgery. No differences were found when analyzing functional parameters and electrophysiological tests, and both groups obtained good analgesia. 16

Like the previous study, Cebesoy et al. 17 divided 40 patients into 2 groups. The non-immobilized group was allowed immediate mobility, while the other group was only allowed movement after 10 days when the immobilization was removed. Levine's Carpal Tunnel Questionnaire (CTQ) was applied preoperatively and in the 1st and 3rd months after surgery. The Symptom Severity Scale (SSS) and the Functional Status Scale (FSS) were applied for this assessment. In the observation after 3 months, it was identified that the group that received only the dressing had lower SSS values. This superiority was attributed to early rehabilitation. Furthermore, 16 patients (80%) complained of discomfort associated with using the splint in the immobilized group. 17

Tinfhofer's group evaluated 63 wrists of 60 patients, comparing dorsal immobilization for 1 week versus a 2-day dressing in the postoperative period of CTS decompression. Follow-up occurred after 3 and 6 months, assessing pain parameters, 2-point discrimination, grip and pinch strength, and electrophysiological studies. The follow-up showed that the individuals in the 2nd group had better grip and pinch strength results at 6 months. 18

Similarly, Shalimar et al. 19 studied 30 patients. Sixteen received immobilization for 1 week, and 14 patients only received bandages. They were evaluated after 1 week, 2 months, and 6 months. The following parameters were observed: visual analog scale (VAS), 2-point discrimination, grip and pinch strength, abductor pollicis brevis strength, and the Boston questionnaire. The two groups had no significant difference in any of the parameters assessed.

A total of 249 patients were divided into three groups: 80 without orthoses, 83 with removable orthoses, and 86 with non-removable orthoses. The following criteria were assessed: Quick Disabilities of the Arm, Shoulder, and Hand (QuickDASH); SSS, and FSS; grip and pinch strength; wrist flexion and extension; and measurement of pain at rest and in action, using the Numerical Pain Rating Scale (NPRS). The outcomes were assessed at 10 to 14 days, 6 weeks, and 3, 6, and 12 months after surgery. The only difference observed was that in the non-removable orthosis group, the pinch strength was lower after 6 and 12 months than that of patients in the other groups. The most common complication was hypersensitivity in the scar area. 20

More recently, in 2023, a published study evaluated 24 patients who underwent endoscopic decompression of CTS. Two groups were randomized, with 12 patients each. In one group, immobilization was used for 2 weeks; in the other, immediate mobilization was allowed. The following parameters were assessed: two-point discrimination test, Semmes-Weinstein monofilament test; occurrence of pain in the pillar; the range of motion of the wrist; Visual Analog Scale (VAS); Boston Carpal Tunnel Questionnaire (BCTQ) score; DASH score; grip and pinch strength; and occurrence of complications. These outcomes were observed 2 weeks and, 1, 2, 3, and 6 months after surgery. ^21^

Two weeks postoperatively, the immobilized group showed better results regarding VAS, grip strength, and pinch, and a lower occurrence of pillar pain. In subsequent comparisons, these differences were not maintained. In two patients in the non-immobilized group, hypersensitivity was observed in the scar in the immediate postoperative period, but this disappeared after 1 month of follow-up. It was, therefore, concluded that in the early follow-up, the immobilized group had better analgesia and grip and pinch strength. However, the two groups were similar at subsequent follow-ups. ^21^

Table 2 shows the RCT articles found.

**Table 2:** Level II evidence studies

Authors	Title	Level of evidence
Finsen et al. 1999	No advantage from splinting the wrist after open carpal tunnel release. A randomized study of 82 wrists	ECR (II)
Bhatia et al. 2000	Does splintage help pain after carpal tunnel release?	ECR (II)
Martins et al. 2006	Wrist immobilization after carpal tunnel release: a prospective study	ECR (II)
Huemer et al. 2007	Postoperative splinting after open carpal tunnel release does not improve functional and neurological outcome	ECR (II)
Cebesoy et al. 2007	Use of a splint following open carpal tunnel release: a comparative study	ECR (II)
Tinhofer et al. 2013	Postoperative care and rehabilitation after open carpal tunnel surgery	ECR (II)
Shalimar et al. 2015	Splinting after Carpal Tunnel Release: Does it really Matter?	ECR (II)
Logli et al. 2018	A Prospective, Randomized Trial of Splinting After Minicarpal Tunnel Release.	ECR (II)
Zhang et al. 2023	The significance of wrist immobilization for endoscopic carpal tunnel release	ECR (II)

### Cross-Sectional Studies - Level of Evidence IV

A survey of members of the American Society for Surgery of the Hand (ASSH) in 1987, by Duncan et al. apud Henry et al. 2 showed that 82% of surgeons immobilized the wrist in the postoperative period of CTS decompression. In 2008, an assessment of 1,091 members of the ASSH showed that this percentage had fallen to 53%. The number of days varied greatly among those interviewed (1–42 days).

Two other evaluations were carried out with ASSH members. In 2012, Leinberry et al. 3 showed that 247 of 659 respondents (around 37%) used postoperative immobilization. In 2014, Munns et al. 4 found that 193 of 710 respondents (around 27%) had this routine.

Table 3 shows the cross-sectional study articles found.

**Table 3:** Level IV evidence studies

Authors	Title	Level of evidence
Henry et al. 2008	Splinting after carpal tunnel release: current practice, scientific evidence, and trends	ET (IV)
Leinberry et al. 2012	Treatment of carpal tunnel syndrome by members of the American Society for Surgery of the Hand: a 25-year perspective	ET (IV)
Munns et al. 2015	Trends in carpal tunnel surgery: an online survey of members of the American Society for Surgery of the Hand	ET (IV)

### Discussion

In the mid-1980s, it was estimated that more than 80% of ASSH members wore a splint postoperatively after open decompression of CTS. Immobilization would theoretically have the advantage of avoiding the bowstring effect on the flexor tendons, wound dehiscence, and median nerve entrapment in the scar, as well as increasing grip and pinch strength and promoting analgesia. However, this practice was already correlated with a longer time to return to daily activities and work. 5 12

In 1978, McDonald et al. apud Henry et al. 2 observed the bowstring effect in 2 patients out of 186 who underwent decompression. Interestingly, in this study, these patients received immobilization in the postoperative period. Another reason would be to avoid prolapse of the median nerve out of the carpal tunnel after sectioning the transverse ligament, observed in 2 cases in 1980 by Inglis. However, early mobility is the best way to prevent friction between the nerve and the overlying skin scar. 2

Due to the scarcity of evidence supporting the use of immobilization, associated with the higher costs related to the practice, 15 it has been observed in recent decades that it has become less and less frequent. Cebesoy et al. 17 showed that it is related to more significant discomfort and making the procedure more expensive without offering any benefits. Other comparative studies advocate early mobility, as it gives a faster recovery in the range of movement. Tinhofer et al. 18 showed the superiority of unrestricted mobility in the immediate postoperative period when analyzing grip strength and pinch parameters in the late postoperative period. On the other hand, much of the evidence also shows that there are no differences between one practice and another. 9 10 11

Among the studies evaluated in this systematic review, only Zhang et al. ^21^ showed that immobilization conferred greater grip and pinch strength and better analgesia in the early postoperative period. However, even in this study, later follow-up showed no difference between applying immobilization or allowing early mobility.

The limitations of this study include the fact that the number of studies with a higher level of evidence (systematic reviews and RCTs) on the subject is still small.

## Discussion

In the mid-1980s, it was estimated that more than 80% of ASSH members wore a splint postoperatively after open decompression of CTS. Immobilization would theoretically have the advantage of avoiding the bowstring effect on the flexor tendons, wound dehiscence, and median nerve entrapment in the scar, as well as increasing grip and pinch strength and promoting analgesia. However, this practice was already correlated with a longer time to return to daily activities and work. 5 12

In 1978, McDonald et al. apud Henry et al. 2 observed the bowstring effect in 2 patients out of 186 who underwent decompression. Interestingly, in this study, these patients received immobilization in the postoperative period. Another reason would be to avoid prolapse of the median nerve out of the carpal tunnel after sectioning the transverse ligament, observed in 2 cases in 1980 by Inglis. However, early mobility is the best way to prevent friction between the nerve and the overlying skin scar. 2

Due to the scarcity of evidence supporting the use of immobilization, associated with the higher costs related to the practice, 15 it has been observed in recent decades that it has become less and less frequent. Cebesoy et al. 17 showed that it is related to more significant discomfort and making the procedure more expensive without offering any benefits. Other comparative studies advocate early mobility, as it gives a faster recovery in the range of movement. Tinhofer et al. 18 showed the superiority of unrestricted mobility in the immediate postoperative period when analyzing grip strength and pinch parameters in the late postoperative period. On the other hand, much of the evidence also shows that there are no differences between one practice and another. 9 10 11

Among the studies evaluated in this systematic review, only Zhang et al. ^21^ showed that immobilization conferred greater grip and pinch strength and better analgesia in the early postoperative period. However, even in this study, later follow-up showed no difference between applying immobilization or allowing early mobility.

The limitations of this study include the fact that the number of studies with a higher level of evidence (systematic reviews and RCTs) on the subject is still small.

## Conclusion

Most of the evidence currently available indicates that the use of immobilization after decompression of CTS is not necessary. However, it is worthwhile carrying out more studies on the subject, as not many publications elucidate this issue.
