# Supplementary material for: Wrist Immobilization after Surgical Decompression of the Median Nerve in Carpal Tunnel Syndrome: A Systematic Review
Source: Rev Bras Ortop (Sao Paulo). 2024 May 19;59(4):e504–9. doi: 10.1055/s-0044-1785665 (PMC11374399; doi:10.1055/s-0044-1785665)
Supplement: Related texts

# Research article: Imobilização do punho após a descompressão cirúrgica do nervo mediano na síndrome do túnel do carpo: Uma revisão sistemática

**Keywords:** nervo mediano, síndrome do túnel carpal, traumatismos do punho, traumatismos dos nervos periféricos

## Abstract

**Objetivo** A neuropatia compressiva mais comum dos membros superiores é a síndrome do túnel do carpo (STC). Historicamente, houve uma tendência à aplicação de imobilização no pós-operatório, prática que tem diminuído nos últimos anos. O objetivo desta revisão é avaliar se existem evidências científicas que justifiquem o uso da imobilização nos cuidados pós-operatórios de descompressão da STC.

**Métodos** Foram utilizadas as seguintes bases de dados: Biblioteca Virtual em Saúde (BVS), PubMed National Library of Medicine (NLM), Cochrane Library, Scientific Electronic Library Online (SciELO), e EMBASE. Foram utilizados os seguintes critérios de inclusão: 1) discussão do pós-operatório de cirurgias de descompressão do nervo mediano na STC; 2) comparação dos resultados, após descompressão cirúrgica, na STC entre imobilização do punho ou apenas curativo local; 3) todos os idiomas, independentemente do ano de publicação; e 4) todos os tipos de publicações. Foram utilizados os seguintes critérios de exclusão: 1) estudos que não avaliaram o pós-operatório de descompressão da STC; 2) falta de avaliação do resultado relacionado à conduta de aplicação de curativo local e/ou alguma forma de imobilização do punho após o procedimento cirúrgico de descompressão; e 3) publicações repetidas

**Resultados** A busca bibliográfica resultou em 336 publicações relevantes. Ao final, foram escolhidas 18 publicações. Foram encontradas revisões sistemáticas, ensaios clínicos randomizados e estudos transversais.

**Conclusões** Devido à escassez de evidências que apoiem o uso da imobilização, associada aos custos mais elevados relacionados com a prática, nas últimas décadas, essa tem se tornado cada vez menos frequente.

**Relevância clínica** Na literatura há descrições de duas abordagens nos cuidados pós-operatórios de descompressão da STC: imobilização ou apenas curativo local. Vale a pena avaliar qual é a melhor entre as duas de acordo com as evidências científicas disponíveis.

## Introdução

A neuropatia compressiva mais comum dos membros superiores é a síndrome do túnel do carpo (STC). O sintoma mais frequente é a parestesia, especialmente à noite, na região inervada pelo nervo mediano. O tratamento cirúrgico é preferível nos casos em que a terapia conservadora falha. A descompressão cirúrgica pode ser via aberta ou endoscópica. 1 Historicamente, havia uma tendência de aplicação de imobilização no pós-operatório, prática essa que tem diminuído nos últimos anos. 2 3 4 A imobilização, teoricamente, teria a vantagem de evitar o efeito arco de corda dos tendões flexores, bem como promoveria analgesia. 5 No entanto, estudos mais recentes não corroboram com essa ideia.

Na literatura, existe descrição de duas condutas no período pós-operatório de descompressão da STC: imobilização ou curativo local apenas. Destarte, é válido que se avalie qual a melhor dentre as duas, de acordo com as evidências científicas disponíveis.

O objetivo é avaliar os desfechos após cirurgias de descompressão do túnel do carpo, quando se imobiliza o punho e quando se faz apenas curativo, e compará-los.

## Materiais e Métodos

## Estratégia de Pesquisa

A busca ativa por artigos na literatura foi realizada em 06 de maio de 2023. A pesquisa utilizou os seguintes bancos de dados: BVS (Biblioteca Virtual em Saúde), PubMed (National Library of Medicine - NLM), Cochrane Library, SciELO (Scientific Eletronic Library Online), EMBASE. Os descritores utilizados, em conjunto com os operadores booleanos, foram: *Carpal Tunnel Syndrome* [Mesh] OR *carpal tunnel syndrome* [tw] OR *carpal tunnel release* [tw] AND *Postoperative Care* [Mesh] OR *postoperative* [tw] OR *postoperative care* [tw] AND *Restraint, Physical* [Mesh] OR *immobilization* [tw] OR *splint* * [tw] AND *Bandages* [Mesh] OR *dress* * [tw] OR bandage* [tw]. Uma Pesquisa de referência cruzada também foi realizada nas bases de dados, com o objetivo de encontrar artigos não identificados inicialmente.

A estratégia de pesquisa em cada banco de dados está detalhada em apêndices ao final do trabalho.

## Critérios de Seleção

Os seguintes critérios foram empregados na inclusão dos estudos: 1) discussão sobre o pós-operatório de cirurgias de descompressão do nervo mediano em STC 2) comparação dos desfechos, após descompressão cirúrgica, em STC, entre imobilização do punho ou apenas curativo local; 3) todas as linguagens, independente do ano de publicação; e 4) todos os tipos de publicações.

Foram elencados os seguintes critérios de exclusão: 1) estudos que não avaliassem o pós-operatório de descompressão de STC; 2) ausência da avaliação do desfecho relacionado à conduta de fazer curativo local e/ou alguma forma de imobilização do punho após o procedimento cirúrgico; e 3) publicações repetidas.

## Coleta de Dados

O processo de coleta de dados foi realizado por dois pesquisadores. Inicialmente, de forma independente, foram analisados os títulos e resumos de cada publicação, sendo em seguida discutidas as inconsistências e obtido um consenso. A posteriori, foi realizada a leitura completa de cada publicação, também de forma independente, sendo realizadas reuniões para decisão de quais estudos deviam serem incluídos e excluídos, chegando-se a um consenso mútuo.

## Nível de Evidência

Os estudos foram classificados de I a V de acordo com o nível hierárquico de evidências. 6 As diretrizes do Preferred Reporting Items for Systematic Reviews and Meta-Analyses (PRISMA) foram usadas nesta revisão para a busca, extração de dados, e análise de resultados. 7

## Resultados

A busca na literatura resultou em 336 publicações relevantes. Após a remoção das duplicatas, foram obtidos 210 artigos. Posteriormente, após análise dos títulos e resumos, 179 estudos foram excluídos, resultando em um total de 31 artigos elegíveis. Após uma leitura completa do texto, quatro artigos foram eliminados pela disponibilidade apenas do título nas plataformas digitais, e outros cinco foram excluídos pela disponibilidade apenas do resumo. Dois artigos foram eliminados por não apresentarem resultados finais (disponibilidade apenas do corpo do trabalho, pois não haviam sido finalizados), e outros dois estudos foram excluídos após leitura completa do texto, por não se adequarem ao objetivo do trabalho. Ao final, foram elencadas 18 publicações. A Fig. 1 ilustra o processo de pesquisa.

**Fig. 1:** Diagrama do *Preferred Reporting Items for Systematic Reviews and Meta-Analyses* (PRISMA), para ilustrar o processo de pesquisa de acordo com os critérios de inclusão e exclusão deste estudo.

## Revisões Sistemáticas - Nível de Evidência I

A revisão sistemática conduzida por Ashworth encontrou estudos clínicos randomizados (ECRs) comparando grupos de imobilização versus não imobilização, após descompressão de STC. Aos 6 meses, não foram encontradas diferenças na força de preensão e pinça entre os pacientes dos 2 grupos. Um outro ECR mostrou que o retorno ao trabalho foi mais lento no grupo imobilizado (27 dias contra 17), bem como apresentou maiores índices de dor de pilar e sensibilidade na cicatriz após 1 mês. 8

Semelhantemente, Huisstede et al. 9 encontraram cinco ECRs que não mostraram evidências que provassem benefício no uso de imobilização. A força de preensão e pinça, analgesia e funcionalidade foram equiparáveis em ambos os grupos.

Uma revisão sistemática realizada por Peters et al., 10 que incluiu 20 ensaios com 1.445 participantes, encontrou pouca evidência científica a favor do uso de imobilização pós-operatória. Posteriormente, em 2015, Peters et al. 11 atualizaram a revisão realizada anteriormente em 2013. Foram incluídos 22 ensaios e 1.521 pacientes. Os resultados continuaram sendo os mesmos.

A Tabela 1 apresenta os artigos de revisão sistemática encontrados.

**Tabela 1:** Estudos de evidência nível I

Autores	Título	Nível de evidência
Ashworth 2010	Sindrome do túnel carpal	RS (I)
Huissttede et al. 2010	Sindrome do túnel carpal. Parte II: eficácia dos tratamentos cirúrgicos - uma revisão sistemática	RS (I)
Peters et al. 2013	Reabilitação após liberação do túnel do carpo	RS (I)
Peters et al. 2016	Reabilitação após liberação do túnel do carpo	RS (I)

## ECR - Nível de Evidência II

Um ECR avaliou 40 pacientes e 43 descompressões abertas de STC, comparando o pós-operatório de 2 semanas entre um grupo de 26 que recebeu tala, e um grupo de 17 que recebeu apenas curativo volumoso. Não foi observado efeito benéfico naquele grupo, ao serem avaliados os parâmetros objetivos de força de preensão, força de pinça, complicações, arco de movimento na mão e nos dedos, e o parâmetro subjetivo de satisfação pessoal. 5

Outro ECR avaliou 50 pacientes, divididos igualmente em dois grupos, sendo que um recebeu uma tala após o procedimento de descompressão para STC, enquanto ao outro grupo foi permitido arco de movimento livre já no pós-operatório imediato. No grupo submetido à imobilização, foram observados maiores índices de dor, sensibilidade na cicatriz, rigidez e maior tempo de recuperação. 12

Em uma publicação, Finsen et al. 13 analisaram 82 punhos submetidos à descompressão de STC, divididos de forma randomizada entre grupo sem imobilização e grupo com imobilização pós-operatória, por 4 semanas. Não foram encontrados benefícios ou malefícios relacionados à prática. Em outra publicação, 52 pacientes foram divididos igualmente em 2 grupos: um de 26 indivíduos que receberam imobilização por duas semanas após descompressão de STC; outro com 26 pacientes sem imobilização pós-operatória. Ambos os grupos apresentaram desfechos semelhantes, incluindo a discriminação em dois pontos. Concluiu-se que o grupo imobilizado não se mostrou superior ao grupo controle. 14

Bhatia et al. 15 mostraram, a partir de um ECR com 102 pacientes, que o uso de uma tala volar no pós-operatório não foi correlacionado a nenhum efeito analgésico na comparação com o grupo controle. Após 50 descompressões de STC via aberta, metade recebeu apenas curativo, e outra metade recebeu uma tala volar por 2 dias. Esses pacientes foram acompanhados até 3 meses após a cirurgia. Não foram encontradas diferenças quando analisados os parâmetros funcionais e os testes eletrofisiológicos, e ambos os grupos obtiveram boa analgesia. 16

Cebesoy et al. 17 dividiram 40 pacientes em 2 grupos, de modo semelhante ao estudo anterior. Foi permitida a mobilidade imediata ao grupo não imobilizado, enquanto ao outro grupo só foram permitidos movimentos após 10 dias, quando a imobilização foi retirada. O *Carpal Tunnel Questionnaire* (CTQ) de Levine foi aplicado no pré-operatório, bem como no 1° e no 3° mês após a cirurgia. Para essa avaliação, duas escalas foram aplicadas: a *Symptom Severity Scale* (SSS) e a *Functional Status Scale* (FSS). Na observação após 3 meses, foi identificado que o grupo que recebeu apenas curativo apresentou valores mais baixos na SSS. Essa superioridade foi atribuída à reabilitação precoce. Outrossim, no grupo imobilizado, 16 pacientes (80%) se queixavam de desconforto associado ao uso da tala. 17

O grupo de Tinfhofer avaliou 63 punhos de 60 pacientes, comparando imobilização dorsal por uma semana versus curativo por 2 dias no pós-operatório de descompressão de STC. O acompanhamento se deu após 3 e 6 meses, avaliando-se os parâmetros de dor, discriminação em 2 pontos, força de preensão e pinça, além de estudos eletrofisiológicos. No seguimento, foi visto que, no tocante à preensão e pinça, aos 6 meses, os indivíduos do 2° grupo apresentaram resultados melhores. 18

Shalimar et al., 19 de modo semelhante, estudaram 30 pacientes. Dezesseis receberam imobilização por uma semana, e 14 pacientes apenas curativos. Eles foram avaliados após 1 semana, 2 e 6 meses, respectivamente. Os seguintes parâmetros foram observados: escala visual analógica (EVA), discriminação em 2 pontos, força de preensão e pinça, força do abdutor curto do polegar, e questionário de Boston. Não houve diferença significativa entre os dois grupos em nenhum dos parâmetros avaliados.

Foram divididos 249 pacientes em 3 grupos: 80 sem órtese, 83 com órtese removível e 86 com órtese não removível. Os seguintes critérios foram avaliados: *Quick Disabilities of the Arm, Shoulder and Hand* (QuickDASH); SSS e FSS; força de preensão e pinça; flexão e extensão do punho; mensuração da dor em repouso e na ação pela *Numerical Pain Rating Scale* (NPRS). Os desfechos foram avaliados de 10 a 14 dias, após 6 semanas, e após 3, 6 e 12 meses da cirurgia. A única diferença observada foi que, no grupo da órtese não removível, após 5 e 12 meses, a força de pinça foi menor do que aquela dos pacientes dos outros grupos. A complicação mais comum foi hipersensibilidade na área da cicatriz. 20

Mais recentemente, no ano de 2023, um estudo publicado mostrou a avaliação de 24 pacientes submetidos à descompressão endoscópica de STC. Dois grupos foram criados aleatoriamente, com 12 pacientes cada um. Em um grupo, foi usada imobilização por 2 semanas, e, no outro, foi permitida a mobilização imediata. Os seguintes parâmetros foram avaliados: teste da discriminação em dois pontos, teste do monofilamento de Semmes-Weinstein; ocorrência de dor no pilar; arco de movimento do punho; EVA; escore *Boston Carpal Tunnel Questionnaire* (BCTQ); escore DASH; força de preensão e pinça; ocorrência de complicações. Esses desfechos foram observados após 2 semanas, e após 1, 2, 3 e 6 meses da cirurgia. ^21^

Após 2 semanas de pós-operatório, o grupo imobilizado apresentou melhores resultados nos quesitos de EVA, força de preensão e pinça, além de menor ocorrência de dor de pilar. Nas comparações subsequentes, essas diferenças não se mantiveram. Em dois pacientes do grupo não imobilizado foi observada hipersensibilidade na cicatriz no pós-operatório imediato, mas que desapareceu a partir de 1 mês de acompanhamento. Concluiu-se, portanto, que no acompanhamento precoce, o grupo que recebeu imobilização apresentou analgesia e força de preensão e pinça melhores. Entretanto, os dois grupos se equipararam nos acompanhamentos subsequentes. ^21^

A Tabela 2 apresenta os artigos de ECRs encontrados.

**Tabela 2:** Estudos de evidência nível II

Autores	Título	Nível de evidência
Finsen et al. 1999	Não há vantagem em imobilizar o pulso após a liberação aberta do túnel do carpo. Um estudo randomizado de 82 pulsos	ECR (II)
Bhatia et al. 2000	A tala ajuda a aliviar a dor após a liberação do túnel do carpo?	ECR (II)
Martins et al. 2006	Imobilização do punho após liberação do túnel do carpo: um estudo prospectivo	ECR (II)
Huemer et al. 2007	A tala pós-operatória após a liberação aberta do túnel do carpo não melhora o resultado funcional e neurológico	ECR (II)
Cebesoy et al. 2007	Uso de tala após liberação aberta do túnel do carpo: estudo comparativo	ECR (II)
Tinhofer et al. 2013	Postoperative care and rehabilitation after open carpal tunnel surgery	ECR (II)
Shalimar et al. 2015	Tala após liberação do túnel do carpo: Isso realmente importa?	ECR (II)
Logli et al. 2018	Um ensaio prospectivo e randomizado de imobilização após liberação do túnel minicarpal	ECR (II)
Zhang et al. 2023	A importância da imobilização do punho para liberação endoscópica do túnel do carpo	ECR (II)

## Estudos Transversais - Nível de Evidência IV

Uma pesquisa realizada com membros da American Society for Surgery of the Hand (ASSH) em 1987, por Duncan et al. apud Henry et al. 2 mostrou que 82% dos cirurgiões imobilizavam o punho no pós-operatório de descompressão de STC. Em 2008, em uma avaliação realizada com 1.091 membros da ASSH, foi visto que essa percentagem caiu para 53%. A quantidade de dias variou bastante dentre os entrevistados (1–42 dias).

Outras duas avaliações foram feitas com membros da ASSH. Em 2012, Leinberry et al. 3 mostraram que 247 de 659 entrevistados (cerca de 37%) utilizavam imobilização pós-operatória. Já em 2014, Munns et al. 4 observaram que 193 de 710 entrevistados (cerca de 27%) tinham essa rotina.

A Tabela 3 apresenta os artigos de estudos transversais encontrados.

**Tabela 3:** Estudos de evidência nível IV

Autores	Título	Nível de evidência
Henry et al. 2008	Talas após liberação do túnel do carpo: prática atual, evidências científicas e tendências	ET (IV)
Leinberry et al. 2012	Tratamento da síndrome do túnel do carpo por membros da Sociedade Americana de Cirurgia da Mão: uma perspectiva de 25 anos	ET (IV)
Munns et al. 2015	Tendências na cirurgia do túnel do carpo: uma pesquisa on-line com membros da Sociedade Americana de Cirurgia da Mão	ET (IV)

## Discussão

Estimava-se, em meados da década de 1980, que mais de 80% dos membros da ASSH usavam, no pós-operatório, uma tala após descompressão aberta de STC. A imobilização teria, teoricamente, a vantagem de evitar o efeito arco de corda nos tendões flexores, deiscência de ferida e encarceramento do nervo mediano na cicatriz, bem como aumentaria a força de preensão e pinça, e promoveria analgesia. Porém já se correlacionava essa prática a um maior tempo para o retorno às atividades do cotidiano e ao trabalho. 5 12

Em 1978, McDonald et al. apud Henry et al. 2 observaram o efeito arco de corda em 2 pacientes, em um total de 186, submetidos à descompressão. Curiosamente, nesse estudo, esses pacientes receberam imobilização no pós-operatório. Outra razão seria evitar o prolapso do nervo mediano para fora do túnel do carpo após secção do ligamento transverso, que foi observado em 2 casos em 1980 por Inglis. No entanto, a melhor maneira de prevenir o atrito entre o nervo e a cicatriz cutânea sobrejacente se mostrou ser a mobilidade precoce.

Devido à escassez de evidências que apoiem o uso da imobilização, associado aos maiores custos relacionados à prática, 15 têm-se visto nas últimas décadas que essa se tornou cada vez menos frequente. Cebesoy et al. 17 mostraram que ela está relacionada a um maior desconforto, além de encarecer o procedimento, sem, contudo, oferecer benefícios. Outros estudos comparativos defendem a mobilidade precoce, pois esta teria a vantagem de conferir uma recuperação mais rápida no arco de movimento. Tinhofer et al. 18 mostraram a superioridade da mobilidade irrestrita no pós-operatório imediato, quando analisados os parâmetros de força de preensão e pinça no pós-operatório tardio. Por outro lado, boa parte das evidências também mostram que não há diferenças entre uma prática e outra. 9 10 11

Dentre os estudos avaliados nessa revisão sistemática, apenas Zhang et al. ^21^ mostraram que, no pós-operatório precoce, a imobilização conferiu maior força de preensão e pinça, bem como melhor analgesia. Entretanto, mesmo nesse estudo, no acompanhamento posterior, foi observado que não havia diferença em se aplicar imobilização ou permitir mobilidade precocemente.

Como limitações do presente trabalho, pode-se citar que a quantidade de estudos de maior nível de evidência (revisões sistemáticas e ECRs) sobre o assunto ainda é pequena.

## Conclusão

A maior parte das evidências disponíveis atualmente apontam que o uso de imobilização no pós-operatório de descompressão de STC não se faz necessário. Entretanto, é válida a realização de uma maior quantidade de estudos sobre o tema, uma vez que não existem muitas publicações que elucidam essa questão.
